# A Luciferase Mutant with Improved Brightness and Stability for Whole-Cell Bioluminescent Biosensors and In Vitro Biosensing

**DOI:** 10.3390/bios12090742

**Published:** 2022-09-09

**Authors:** Maria Maddalena Calabretta, Denise Gregucci, Héctor Martínez-Pérez-Cejuela, Elisa Michelini

**Affiliations:** 1 Department of Chemistry “Giacomo Ciamician”, Alma Mater Studiorum-University of Bologna, Via Selmi 2, 40126 Bologna, Italy; 2 Center for Applied Biomedical Research (CRBA), IRCCS St. Orsola Hospital, 40138 Bologna, Italy; 3 CLECEM Group, Department of Analytical Chemistry, University of Valencia, C/Dr. Moliner, 50, 46100 Burjassot, Valencia, Spain; 4 Health Sciences and Technologies Interdepartmental Center for Industrial Research (HSTICIR), University of Bologna, 40126 Bologna, Italy

**Keywords:** bioluminescence, luciferase, whole-cell biosensors, biosensing, ATP, NF-kB

## Abstract

The availability of new bioluminescent proteins with tuned properties, both in terms of emission wavelength, kinetics and protein stability, is highly valuable in the bioanalytical field, with the potential to improve the sensitivity and analytical performance of the currently used methods for ATP detection, whole-cell biosensors, and viability assays among others. We present a new luciferase mutant, called BgLuc, suitable for developing whole-cell biosensors and in vitro biosensors characterized by a bioluminescence maximum of 548 nm, narrow emission bandwidth, favorable kinetic properties, and excellent pH- and thermo-stabilities at 37 and 45 °C and pH from 5.0 to 8.0. We assessed the suitability of this new luciferase for whole-cell biosensing with a cell-based bioreporter assay for Nuclear Factor-kappa B (NF-kB) signal transduction pathway using 2D and 3D human embryonic kidney (HEK293T) cells, and for ATP detection with the purified enzyme. In both cases the luciferase showed suitable for sensitive detection of the target analytes, with better or similar performance than the commercial counterparts.

## 1. Introduction

Bioluminescence (BL) is the emission of light occurring in living organisms, including bacteria, fungi, various insects and marine organisms, as a result of a chemical reaction catalyzed by an enzyme, called luciferase, which oxidates a substrate, generally referred to as luciferin, in the presence of molecular oxygen and eventually cofactors, such as ATP as in the case of firefly luciferases [1,2,3].

The most extensively studied enzyme is luciferase from the North American firefly *Photinus pyralis* (PpyLuc), which catalyzes a two-step reaction using the substrate D-luciferin (D-LH_2_), ATP, and molecular oxygen to yield oxyluciferin in an electronically excited state. A yellow−green emission (λmax = 560 nm at pH 7.8) is observed when the excited oxyluciferin relaxes to the ground state. Differently from fluorescence, BL does not require any excitation light source and BL background levels in the cellular environment are extremely low, resulting in very high signal-to-noise ratios, thus being a formidable tool for BL in vivo imaging, especially considering red-emitting reporters [4,5]. Thanks to the high quantum yield (about 40%) of PpyLuc-catalyzed BL, luciferase can be quantified at attomole levels using photomultiplier tubes or charge-coupled devices [6]. Since the cloning of the first luciferase in the 1970s [7], the light-emitting reaction has been investigated together with factors affecting the emission wavelength, kinetics and other properties of the enzyme [8,9].

Nowadays a wide portfolio of BL luciferase enzymes has been obtained by cloning the corresponding genes from marine and terrestrial organisms [10,11].

The availability of new BL proteins relying on diverse biochemical reactions with tuned properties, both in terms of emission wavelength, kinetics and protein stability, and requiring different BL substrates, is highly valuable in the bioanalytical field, with the potential to improve the sensitivity and analytical performance of currently used methods, including ATP detection [12], whole-cell and cell-free biosensors [13] and viability assays, paving the way to new assay formats even in miniaturized devices [14,15,16]. In addition exploiting both random and site-directed mutagenesis, a number of luciferases with altered emission properties (e.g., shifted emission wavelengths, higher quantum yield emission, longer kinetics) were obtained, providing an untapped source of bioanalytical tools suitable for in vitro biosensing and in vivo imaging [17,18]. In fact, all these properties make them a powerful bioanalytical tool for unravelling molecular pathways involved in the etiopathogenesis of several diseases, for tracking molecules, cells, and even for monitoring protein–protein interactions [19,20,21,22,23]. In addition, thanks to the availability of highly sensitive light detectors, including CCD (Charged-Coupled Device), CMOS (Complementary Metal Oxide Semiconductor), PMT (Photomultiplier tubes) and SiPM (Silicon Photomultipliers), several assays were turned into portable biosensors which were successfully applied for the on-site analysis of pharmaceutical, environmental, forensic, and food samples [24,25,26,27,28].

However, practical applications of luciferase-based biosensors for on-field applications are hampered by the delicate nature of the enzyme which is denatured in harsh conditions (e.g., high temperature, non-optimal pH, presence of chemicals) [29]. To this end several strategies have been explored for enhancing the stability and the catalytic activity [30,31], or to obtain thermostable and pH-resistant mutants and variants emitting at different wavelengths [32,33,34,35].

Here we report a new PpyLuc luciferase mutant (BgLuc) characterized by improved brightness and stability useful for the design of in vitro and whole-cell BL biosensors. We compared the physical and spectral properties of the new luciferase protein with the commercial luciferase Luc2 and demonstrated the potential of BgLuc for in vitro ATP biosensing. We also compared the performance of BgLuc with the Luc2 gene in human embryonic kidney (HEK293T) cells. As proof of concept, to preliminary assess the suitability of BgLuc as a reporter protein, 2D and 3D cell-based assays for the evaluation of (anti)-inflammatory activity were developed by transfecting HEK293T cells with a plasmid encoding for BgLuc under the regulation of NF-kB response element. The results confirmed its suitability for sensitive detection of the target analytes, with better or similar performance than the commercial counterparts.

## 2. Materials and Methods

### 2.1. Chemical and Reagents

*Escherichia coli* (*E. coli*) JM109 competent cells for plasmid propagation and the SOC medium (tryptone 20 g/L, yeast extract 5 g/L, NaCl 5.0 M 2 mL/L, KCl 1.0 M 2.5 mL/L, MgCl_2_ 1.0 M 10 mL/L, MgSO_4_ 1.0 M 10 mL/L, D-Glucose 1.0 M 20 mL/L) were from Sigma (St. Louis, MO, USA), while *E. coli* BL21 competent cells for protein expression were from Agilent Technologies (Santa Clara, CA, USA). Luria-Bertani (LB) medium and LB-Agar plates used for cell cultures were prepared whit Select Agar and LB (Lennox L Broth) from Sigma (St. Louis, MO, USA) added with ampicillin (50 μg/mL). All media and materials were autoclaved for 20 min at 121 °C.

Human embryonic kidney (HEK293T) cells were from ATCC (American Type Culture Collection [ATCC], Manassas, VA, USA) and materials used for culturing of cells were from Carlo Erba Reagents (Cornaredo, Milano, Italy). The enzymes required for cloning were from Thermo Fisher Scientific (Waltham, MA, USA). The kits for plasmid extraction, the mammalian expression plasmid pGL4.32[luc2P NF-kB-RE Hygro], beetle luciferin potassium salt (D-luciferin) and BrightGlo substrate were from Promega (Madison, WI, USA). Protino Ni-IDA Resin and 14 mL Protino Columns required for protein extraction were purchased from Macherey-Nagel GmbH and Co. KG (Düren, Germany). The new *P. pyralis* luciferase mutant BgLuc gene was synthesized by Eurofins Genomics (Ebersberg, Germany). The reporter vectors pCDNA_BgLuc and pGL4.32[NF-κB-RE]-BgLuc were obtained by standard molecular cloning procedures). All other chemicals were purchased from Sigma (St. Louis, MO, USA).

### 2.2. Plasmid Construction

The sequence of the *P. pyralis* luciferase mutant, called BgLuc, contains the following mutations F14R, L35Q, V182K, I232K, F465R, Y33N, T214A, A215L, F295L, E354K, V241I, G246A, F250S, N119G and N50D, was synthesized by Eurofin MWG Operon (Ebersberg, Germany) with codon optimization for human expression. The sequence was cloned into pQE-30 UA plasmid (Qiagen) and into pcDNA3.1 (+) vector backbone (Invitrogen, Waltham, Massachusetts, USA) by mean of a blunt ligation, obtaining plasmids pQE-BgLuc and pCDNA-BgLuc. BgLuc was also cloned into pGL4.32[luc2P NF-kB-RE Hygro] (Promega) to replace Luc2P. The obtained plasmid was named pGL4.32[NF-κB-RE]-BgLuc. All constructs were verified by DNA sequencing.Software UCSF ChimeraX was used for luciferase visualization (UCSF ChimeraXStructure visualization for researchers, educators, and developers [36].

### 2.3. Expression and Purification of Luciferase Mutants

Expression plasmid pQE-BgLuc was transformed in BL21 competent cells for luciferase expression and purification as previously described with slight modifications [9]. Briefly, 5 mL cultures were grown in LB medium with 50 µg/mL ampicillin at 37 °C overnight and used to inoculate 250 mL cultures (1:100 dilution), grown at 37 °C with shaking until an OD600 nm of 0.6 was reached. Then cultures were induced with 0.1 mM IPTG (Isopropyl β- D-1-thiogalactopyranoside) and incubated with shaking for 5 h at 30 °C. Bacterial cells were then transferred to 50 mL centrifugation tubes, pelleted by centrifugation at 3000× *g*, 4 °C for 20 min, and stored at −80 °C. Cell-lysis-extraction buffer solution was prepared with 10 μL of lysozyme (10 mg/mL in PBS) and 1 μL of serine protease, PMSF (phenylmethylsulfonyl fluoride) 100 mM in EtOH per 1 mL of B-PER Reagent. The washing solution was LEW Buffer, 300 mM NaCl and 50 mM NaH_2_PO_4_·H_2_O at pH 8.0. Elution Buffer was LEW Buffer plus 250 mM imidazole solution, adjusted to pH 8.0. The bacterial pellet was resuspended on ice using 2 mL of cell-lysis-extraction buffer. Suspension was incubated for 20 min in ice with gentle resuspension every 5 min. A 4 mL volume of LEW Buffer was added to the cell lysate and kept at room temperature for 10 min with continuous resuspension. Ultracentrifugation at 4500× *g*, at 4 °C for 30 min was performed and the clear supernatant was then collected to proceed with a protocol for purification under native using Protino Ni-IDA Resin for purification of his-tag proteins according to the Manufacturer’s instructions.

The 300-µL aliquots were eluted in Elution Buffer and protein concentration was determined by a Bio-Rad procedure using bovine serum albumin as the standard. The activity of the purified proteins was evaluated using a luminometer (Thermo Scientific Varioskan LUX Multimode microplate reader) using 4 µL of eluted protein, 100 µL of phosphate-buffered saline (PBS), and 100 µL of BrightGlo Luciferase Assay System (Promega).

### 2.4. Heat Inactivation Studies

BgLuc luciferase (0.08–0.1 mg) was incubated at 37 °C in 0.2 mL of 25 mM glycylglycine buffer (pH 7.8). Aliquots (4 μL) were taken at regular intervals (each 10 min and after an overnight incubation) to track enzyme inactivation. Half-life was calculated using first-order rate constants obtained from log plots of percentage activity remaining versus time.

### 2.5. Luciferase Emission Spectra, Thermal and pH Stability Studies

BgLuc BL emission spectrum was obtained in a white 384 well plate at 37 °C using the Thermo Scientific Varioskan LUX Multimode Microplate Reader using 10 μL of the BgLuc purified enzyme in Elution Buffer and 10 μL of BrightGlo substrate. Emission spectra were also obtained after 30 min incubation of the enzyme either at 37 °C or 45 °C.

Aliquots (15 μL) of the purified BgLuc luciferase (0.6 mg/mL) were transferred to thin-walled PCR tubes and incubated for 30 min at temperatures ranging from 37 to 45 °C in triplicate using a thermal cycler (T100, Bio-Rad). Luciferase activity was measured using the Thermo Scientific Varioskan LUX Multimode Microplate Reader after the addition of D-Luciferin substrate 1.0 mM in HEPES 0.1 M. The incubated BgLuc enzyme solutions (10 μL) were mixed with 1.0 mM D-luciferin solution in Hepes (2.5 μL) in a white 384-well microplate, 15 μL of Lew buffer and MgCl_2_ (10 mM). Luciferase reaction was started by injecting 5 μL of ATP solution (20 mM), and the emission spectra were recorded 1 min after injection. The final concentrations in the reaction were 2.5 mM MgCl_2_, 9.0 μg/mL BgLuc enzyme, 2.0 mM ATP, and 40 μM D-luciferin. Heat inactivation studies were also repeated using the commercial BrightGlo substrate with a ratio of 1:1.

BL emission spectra were obtained at pH 5.0, 7.0 and 8.0 as previously described by [37]. The pH values of the reaction mixtures were confirmed before and after all spectra were obtained. Spectral measurements at 37 °C were obtained after the addition of the luciferases to the buffered reagent solutions maintained at 37 °C.

### 2.6. Determination of Kinetic Parameters and ATP Detection

Luciferase activity was assessed with varying concentrations of ATP (from 2.0 × 10^−6^ to 2.0 × 10^1^ mM) and excess of D-luciferin (1.0 mM) in the presence of the enzyme (3 μg) for calculating the apparent Km for ATP. For D-luciferin Km, 3 μg of luciferase were used with 1.0 mM ATP and D-Luciferin concentrations in the concentration range from 1.0 × 10^−3^ to 5.0 mM. All measurements were performed by measuring light intensity 20 min after the start of the reaction at 25 °C.

Each measurement was performed in duplicate, and experiments were performed at least three times. The data were fitted to the Michaelis−Menten equation using the GraphPad Prism v8.3.0 software (GraphPad Software, La Jolla, CA, USA) to calculate apparent Km values.

### 2.7. Whole-Cell Biosensor for Inflammation Activity

To characterize BgLuc luciferase, one day before transfection, HEK293T cells were plated in black, clear bottom 96-well (2.0 × 10^4^ cells per well). The day after seeding, cells were transfected with 0.11 μg of pcDNA-BgLuc according to the manufacturer’s instructions, using a FuGENE HD/DNA ratio of 3:1 and incubated at 37 °C with 5% CO_2_. The same procedure was carried out for pCDNALuc2. After 24 h post-transfection, emission spectra were recorded with a luminometer (Thermo Scientific Varioskan LUX Multimode Microplate Reader) after injection of 100 µL of 1.0 mM D-luciferin citrate solution pH 5.0 or 50 µL BrightGlo substrate.

To monitor the inflammatory pathway activation, HEK293T cells were plated in 96-well microspace round bottom cell culture plates (Corning^®^ Elplasia^®^ Plates) at a concentration of 2.0 × 10^4^ cells per well as previously described [38] and transiently transfected with plasmid encoding for BgLuc under the regulation of NF-kB responsive element (pGL4.32[NF-κB-RE]-BgLuc). 24 h post-transfection, spheroids were treated in triplicate with 50 µL of TNFα solutions in culture medium (0.1–10 ng/mL) or with 50 µL of culture medium as a control. After 5 h incubation at 37 °C, 50 µL BrightGlo substrate was added to each well. The same procedure was carried out using the commercial reporter vector pGL4.32[luc2P NF-kB-RE Hygro]. The half maximal effective concentration (EC_50_), which is the concentration of the inducer (TNFα) which produces 50% of the maximum possible response, was calculated using the following equation:

Y = Bottom + (Top − Bottom)/(1 + 10^((LogEC50 − X) × Hillslope))

where X is the logarithmic concentration of TNFα.

## 3. Result and Discussion

A new firefly luciferase mutant, named BgLuc, has been designed to obtain a stable and bright mutant that can be applied for in vitro and in vivo biosensing applications. Fifteen aminoacid changes were introduced into the PpyLuc coding sequence to provide activity enhancement and protection against red-shifting of BL at low pH and temperature stability (Figure S1, Supplementary Materials).

To improve both pH and thermo-stability, firstly we mutated the *P. pyralis* luciferase gene by introducing previously reported mutations which showed able to increase the BL emission of the wild-type luciferase at pH 6.0 and improve the stability at higher temperatures (up to 45 °C). Since these mutations (F14R, L35Q, V182K, I232K, F465R) showed additive effects and did not affect the active site, Law et al. reported no changes in the Km for ATP and D-LH_2_, while providing higher enzyme stability due to improved resistance to conformational change [39]. The increased light production probably is the result of stabilizing charge–charge interactions due to the addition of positively charged groups at positions 14, 182 and 465 that cause, presumably, an higher local negative charge density. To increase structural stability by establishing more favorable local interactions and inspired by the same rational of mutating solvent-exposed hydrophobic amino acids to hydrophilic residues, an additional mutation, Y33N, not yet reported, was also included. The three mutations T214A, A215L, and F295L, were also added to improve both in vitro stability and in vivo sensitivity [40]. Mice inoculated with tumor cells stably transfected with mutant luciferases carrying these mutations showed increased light output when compared to mice inoculated with tumors expressing the wild-type luciferase. In addition, to further improve the pH and thermostability of the luciferase the following mutations E354K, V241I, G246A, and F250S, were also introduced [9,41], together with N119G and N50D, mutations also present in the Luc+ gene in pGL3 vectors (Promega). Since the mutations were not introduced one by one, the presence of counteracting, additive or synergistic effects were not evaluated, and it will be the subject of investigation in further studies.

**Figure 1:**
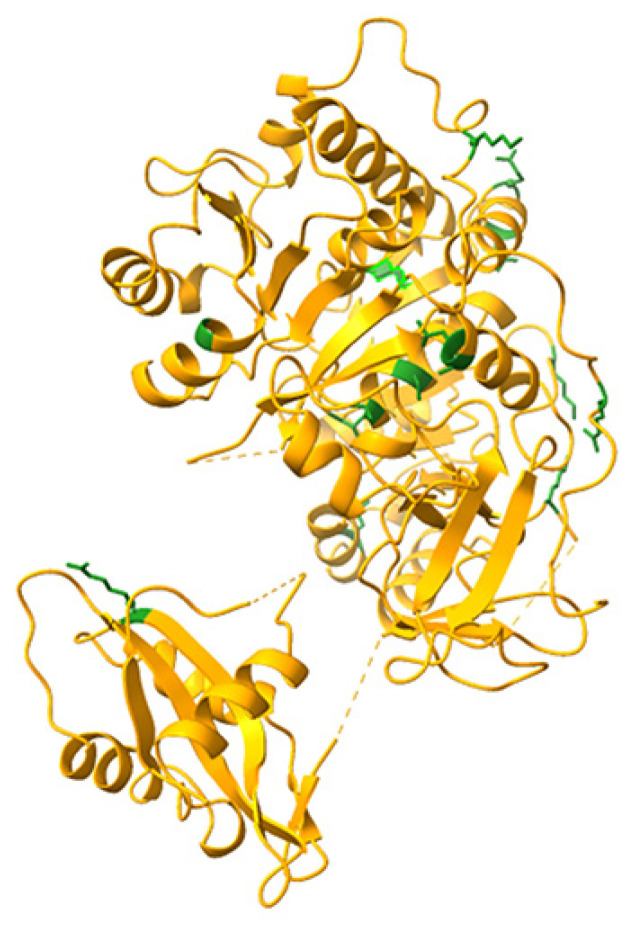
Locations of the mutated aminoacids, shown in green, depicted in the Ppy luciferase crystal structure (Protein Data Bank:1LCI). Modelling was performed with UCSF ChimeraX.

The relative positions of the mutated aminoacids in the crystal structure of PpyLuc (PDB 1LCI) are shown in Figure 1.

### 3.1. Luciferase Characterization: Bioluminescence Emission Kinetics and Spectra

**Figure 2:**
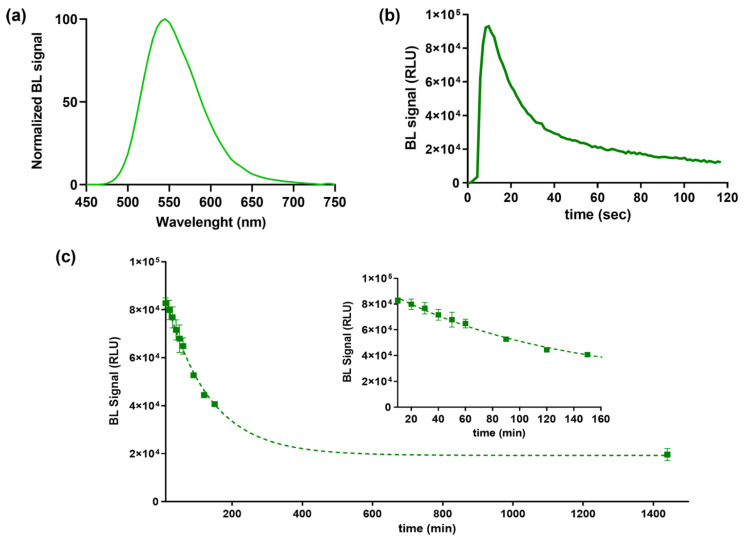
(**a**) Normalized BL emission spectrum, (**b**) emission kinetic and (**c**) heat inactivation studies of purified BgLuc luciferase mutant incubated at 37 °C obtained with Thermo Scientific Varioskan LUX Multimode Microplate Reader. Inset shows the BgLuc heat inactivation study tested until 50% activity loss.

BL emission kinetic and spectra for the purified BgLuc protein were determined using saturating levels of D-LH_2_ (1.0 mM) and Mg–ATP (2.0 mM) at pH 7.8. BgLuc showed a BL emission spectrum with a maximum at approximately 548 nm and half bandwidth of 75 nm (Figure 2a). Kinetic measurements showed a flash-type emission typical of firefly luciferases with a peak after 9.60 s and a signal half-life of 25 s (Figure 2b). The flash height-based measurements relate the maximum achievable overall reaction rate of light emission, a process dependent on the adenylation of substrate firefly luciferin followed by multi-step oxidation of the intermediate to yield the light-emitting species oxyluciferin.

**Figure 3:**
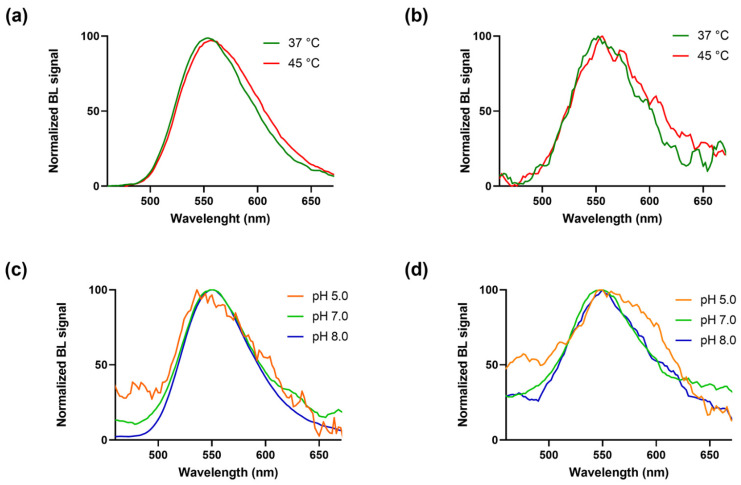
BgLuc mutant emission spectra were obtained at different temperatures with the (**a**) BrightGlo and (**b**) D-Luciferin 1.0 mM substrates and at different pH with the (**c**) BrightGlo and (**d**) D-Luciferin 1.0 mM substrates.

Heat inactivation studies were performed by incubating aliquots of purified protein at 37 °C, luminescence measurements were taken in duplicate every 10 min and after overnight incubation to monitor luciferase activity (Figure 2c). As reported in Table 1, we observed a greater thermostability of this luciferase compared to PpyLuc (half-life of 2.5 h vs. 20 min at 37 °C) [42] and similar to Luc2 (2.5 h vs. 3.0 h at 37 °C) [43] and the lack of emission color change at varying pH (Figure 3), as opposed to the well-known red-shifting at pH 6.0 reported with both PpyWT [42] and Luc2 [44]. Although the BgLuc intensity at pH 5.0 was significantly low, the absence of red-shifting is highly valuable especially for reporter assays with mammalian cells where acidic pH conditions are common due to cell metabolism. The selection of a pH-insensitive luciferase prompts the implementation of a second reporter, i.e., a red-emitting luciferase, used as control or to multiplex the assay. The half-life of BgLuc luciferase is slightly shorter compared to luciferase from other species and also with respect to other firefly luciferase mutants, however after an overnight incubation at 37 °C, remaining luciferase activity was 22%, which is a promising result for its application as reporter protein and for implementation into biosensing platforms (Figure 2c).

### 3.2. Thermal and pH Stability Measurements of BgLuc

A significant drawback for bioanalytical applications that exploit BL is the sensitivity of luciferase to thermal and pH variations. In fact, in an ideal dual-color BL reporter system, where a green and a red-emitting mutant are used to track different targets within living cells grown at 37 °C or expressed in in vivo models such as laboratory animals, the emission spectra of the two signals would not overlap. However, several firefly wild-type luciferases show a significant red-shift when expressed at 37 °C. The introduction of amino acid mutations can overcome this issue to minimize spectral overlap, to obtain narrower bandwidths and very well-separated emission maxima.

Since PpyLuc shows a significant red-shifted emission at 37 °C, thermostability studies of the synthetic BgLuc luciferase were performed, incubating the enzyme for 30 min at different temperatures (37 and 45 °C). Activity of the luciferase samples were then measured in the presence of D-LH_2_ (1.0 mM) and ATP (2 mM) or the commercial BrightGlo substrate (Table 2).

As expected, BgLuc displayed high thermostability (Figure 3a,b), retaining ~85% activity after 30 min at 45 °C vs. the completely inactivation of WT luciferase [30]; this represents an important key point in the perspective of using BgLuc for on-field biosensing applications in which the biosensor must be stable both during transportation and on-site storage; such high temperatures are easily reached in remote areas, especially in those countries with harsh climate conditions.

To evaluate the stability of BgLuc and the emission wavelength at three different pH values, emission spectra were also determined at pH 5.0, 7.0 and 8.0 using the Thermo Scientific Varioskan LUX Multimode Microplate Reader in the presence of D-LH_2_ (1.0 mM) and ATP (2 mM) or the commercial BrightGlo substrate (Table 3).

BgLuc showed overlapping spectra at pH 5.0, 7.0 and 8.0 with a peak at approximately 550 ± 6 nm, and a half bandwidth of 48, 72 and 70 nm, respectively with commercial Brightglo substrate (Figure 3c), and of 124, 94 and 97 nm with D-LH_2_ (1.0 mM) (Figure 3d). Using the same concentration of the BgLuc enzyme in both conditions of pH and temperature, signal intensities obtained with the home-made luciferin substrate (D-LH_2_) were lower than those obtained with the commercial BrightGlo, thus producing spectra with a low signal-to-noise ratio, as in the case of Figure 3b,d, and the spectrum obtained at pH 5.0 with the BrightGlo (Figure 3c). Figure S3 shows the non-normalised spectra with the emissions in RLUs.

It has been previously observed that many thermostable luciferase mutants show a lack of red-shift at acidic pH [45]; this suggests that the red-shift observed at acidic pH could be related to certain conformational changes in the luciferase able to change the characteristics of the active site and to the mutations that confer thermostability [39].

### 3.3. Measurement of Kinetic Parameters and ATP Detection

BgLuc activity in the presence of varying concentrations of D-luciferin (from 1.0 × 10^−3^ to 5.0 mM) was measured in the presence of the enzyme (3 µg) and excess ATP (2 mM) at 25 °C. The light intensity was measured 20 min after the start of the reaction during the glow phase of the luciferase light output in order to obtain a more representative value than the pseudo-steady state of the luciferase reactions relevant to bioimaging applications. Michaelis−Menten equation was used to calculate the apparent Km value of 50 ± 13 µM for D-LH_2_, which is about 3-fold higher than the value of PpyWT [38]. At the same time apparent Km for ATP was similar to that of PpyLuc (21 ± 5 µM vs. 86 ± 7 µM, respectively).

**Figure 4:**
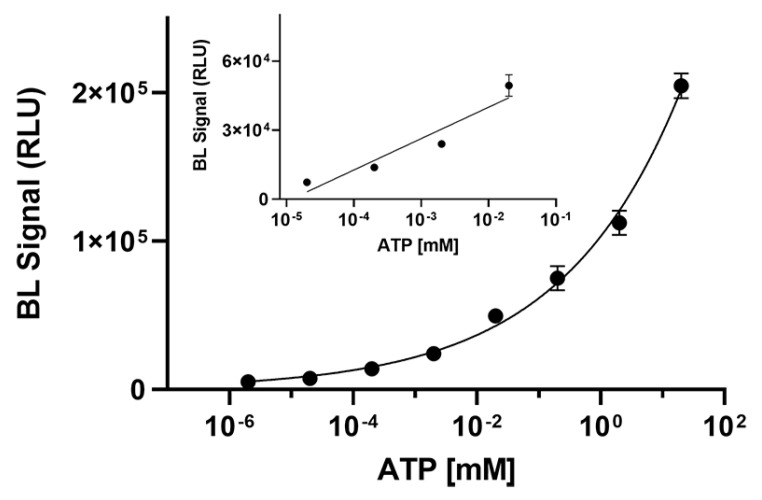
Calibration curve for ATP detection obtained with BgLuc luciferase (6 μL of a 0.5 mg/mL solution). The measurements were performed in triplicate and repeated at least three times.

To demonstrate the suitability of BgLuc luciferase for practical application in ATP detection, an ATP calibration curve (from 2 × 10^−6^ to 20 mM) was obtained and a limit of detection (LOD) of 1.0 nM was obtained corresponding to 6 femtomoles of ATP (Figure 4). As concerns the linear range, a linear correlation in the concentration range of 7.0 × 10^−5^ to 1.3 × 10^−2^ mM for ATP was obtained (R^2^ = 0.9068).

### 3.4. Whole-Cell Biosensor for Inflammation Activity

The enhanced activity of about 35% than PpyLuc, the absence of red-shifting of BL at low pH (~5.0), and improved thermostability make BgLuc an excellent candidate for BL reporter assays and biosensors, in which either high temperatures (i.e., 37 °C for cell culture conditions or during shipping without a strict cold-chain) or low pH conditions (e.g., as a consequence of cell metabolism or when analyzing complex biological samples) are commonly encountered.

To evaluate the potential suitability of BgLuc luciferase for 2D and 3D cell-based assays firstly we characterized BgLuc expression in terms of emission spectra and, as expected, we did not observe significant changes in the emission spectra obtained in 3D cell models when compared with those obtained with HEK293T monolayer cultures (data not shown). We also compared the emission spectra obtained with HEK293T transiently transfected with BgLuc and Luc2 (Figure S2) obtained with D-luciferin and with BrightGlo substrate. No change in red-shifting in emission spectra was observed with the BgLuc luciferase (λmax = 560 nm), confirming its suitability for dual luciferase applications. On the contrary, as previously reported by us, Luc2 shows a marked red-shifting using D-luciferin (λmax = 623 nm), less pronounced (λmax = 608 nm) with the lysing Bright-Glo™ commercial substrate [46].

The suitability of BgLuc as a reporter protein for 3D cell-based assays is of vital importance since these assays provide highly valuable information and reliable bioactivity data thanks to the 3D environment that faithfully mimics in vivo physiological conditions [47].

**Figure 5:**
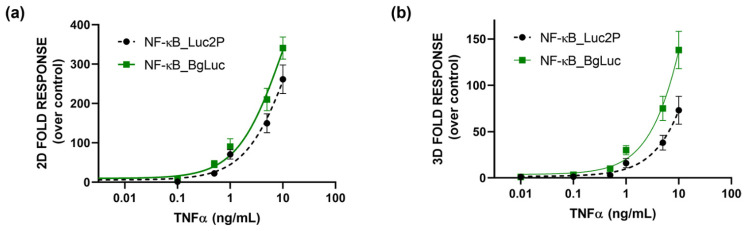
Dose-response curves obtained in (**a**) 2D or (**b**) 3D cell-based assays. HEK293T cells were incubated for 5 h at 37 °C with the TNFα, using BgLuc (solid line) and commercial Luc2P (dotted line) as reporters under the control of NFkB -response element. BL measurements were obtained after the addition of BrightGlo substrate as described in the Materials and Methods section. The experiment was performed in triplicate and repeated at least three times.

As a proof-of-concept, to preliminary assess the suitability of BgLuc as reporter protein, a cell-based assay for the evaluation of (anti)-inflammatory activity was developed by transfecting HEK293T cells with a plasmid encoding for BgLuc under the regulation of NF-kB response element. To evaluate the feasibility of using BgLuc reporter for upgrading 2D drug screening or in vitro biosensing, we developed a 3D assay for inflammatory activity using one-day-old HEK293T spheroids, previously transfected with a reporter construct in which the BgLuc luciferase is placed under the control of the NF-kB response element, and incubated with different concentrations of TNF α (concentration range 0.1–10 ng/mL) for 5 h. The binding of TNFα to its specific endogenous receptor (TNFR) leads to the activation of the intracellular inflammatory pathway with BgLuc expression. Dose-response curves for TNFα were obtained with both monolayer cultures (Figure 5a) and spheroids (Figure 5b), obtaining EC_50_ values of 10.1 ng/mL and 35.2 ng/mL, respectively. In agreement with previous reports by others [48] and us [38,46] a higher NF-kB basal activation (about 3 fold) was found in 3D spheroids than the 2D format; this is probably due to the presence of an intra-spheroid cytokine signaling that induces JNK and NFkB pathways. The results were compared with that obtained with the Promega commercial plasmid reporter (EC_50_ values of 15.3 and 60 ng/mL for 2D cultures and spheroids, respectively) confirming that the BgLuc enzyme has great potential as a BL reporter and in imaging applications. Because PpyLuc has proven to be suitable for dual reporter and cell sensor assays, we expect that BgLuc could perform as well or better because this enzyme produces brighter signals, has similar BL emission which is maintained at low pH and is more stable at 37 °C. The replacement of Luc2 with BgLuc could thus improve the sensitivity of in vitro assays and biosensing applications, such as high-throughput drug screening or ATP biosensors.

## 4. Conclusions

By combining the characteristics of several previously investigated mutations, we designed a new firefly luciferase mutant (BgLuc) with higher activity and improved pH- and thermal stability. The obtainment of new luciferases with improved properties, such as high thermostability, pH-independent emission and the tuned emission wavelength is highly demanded both for increasing assay sensitivity of bioanalytical applications in mammalian cells and for in vitro ATP analysis. The lack of emission color change at low pH and the high thermostability of the new BgLuc luciferase compared to PpyLuc wild type luciferase (2.5 h vs. 20 min at 37 °C) support its suitability for implementation into whole-cell biosensors, for dual luciferase applications and in vitro biosensors. Preliminary bioanalytical applications have been reported corroborating the use of this new luciferase for ATP detection and as a reporter protein for developing bioluminescence cell-based assays.

## Figures and Tables

**Table 1:** Comparison of BL emission properties and half-life of commercial luciferases and BgLuc mutant.

Luciferase	Organism	Emission λmax (nm) (pH 7.8)	Half-Life (h, 37 °C)	Notes	Ref.
PpyWT	*P. pyralis*	557	0.3	pH-sensitive	[42]
Luc2	*P. pyralis*	557	3.0	pH-sensitive	[43,44]
BgLuc	*Synthetic*	548	2.5	pH-independent	

**Table 2:** λmax and half bandwidth of BgLuc mutant at different temperatures (37 °C and 45 °C).

BgLuc Mutant	BL Emission (37 °C)		BL Emission (45 °C)	
	λmax (nm)	Half Bandwidth (nm)	λmax (nm)	Half Bandwidth (nm)
BrightGlo substrate	552	76	556	82
D-LH_2_ substrate	552	78	556	90

**Table 3:** λmax and half bandwidth of BgLuc at varying pH conditions.

BgLuc Mutant	BL Emission pH 5.0		BL Emission pH 7.0		BL Emission pH 8.0	
	λmax (nm)	Half Bandwidth (nm)	λmax (nm)	Half Bandwidth (nm)	λmax (nm)	Half Bandwidth (nm)
BrightGlo substrate	550	48	548	72	552	70
D-LH_2_ substrate	548	124	550	94	550	97

## Data Availability

Not applicable.

## Supplementary Materials

The following supporting information can be downloaded at: https://www.mdpi.com/article/10.3390/bios12090742/s1, Figure S1: Amino acid alignment of the BgLuc and PpyLuc luciferases; Figure S2: Emission spectra of (a) BgLuc and (b) Luc2 luciferase in HEK293T cells obtained with D-LH_2_ in citrate buffer (1.0 mM, pH 5.0) and BrighGlo substrates; Figure S3: BgLuc mutant emission spectra obtained (a) at different temperatures with the BrightGlo and (b) at different pH with the commercial BrightGlo substrate.
