# Supplementary material for: A Luciferase Mutant with Improved Brightness and Stability for Whole-Cell Bioluminescent Biosensors and In Vitro Biosensing
Source: Biosensors (Basel). 2022 Sep 9;12(9):742. doi: 10.3390/bios12090742 (PMC9496056; doi:10.3390/bios12090742)
Supplement: Supplementary file 1 [file biosensors-12-00742-s001.zip › biosensors-1879366-supplementary.pdf]

Supplementary materials

A Luciferase Mutant with Improved Brightness and Stability for Whole-Cell Bioluminescent Biosensors and *In Vitro* Biosensing

Maria Maddalena Calabretta [1,2], Denise Gregucci [1,2], Héctor Martínez-Pérez-Cejuela [1,3] and Elisa Michelini [1,2,4,*]

1 Department of Chemistry "Giacomo Ciamician", Alma Mater Studiorum-University of Bologna, Via Selmi 2, 40126 Bologna, Italy
2 Center for Applied Biomedical Research (CRBA), IRCCS St. Orsola Hospital, Bologna, 40138, Italy
3 CLECEM Group, Department of Analytical Chemistry, University of Valencia, C/Dr. Moliner, 50, 46100, Burjassot, Valencia, Spain
4 Health Sciences and Technologies Interdepartmental Center for Industrial Research (HSTICIR), University of Bologna, 40126 Bologna, Italy
* Correspondence: elisa.michelini8@unibo.it; +39 051 20 9 9533

```
        1                                                                                82
PpyLuc  MEDAKNIKKGPAPFYPLEDGTAGEQLHKAMKRYALVPGTIAFTDAHIEVNITYAEYFEMSVRLAEAMKRYGLNTNHRIVVCS
BgLuc   MEDAKNIKKGPAPRYPLEDGTAGEQLHKAMKRNAQVPGTIAFTDAHIEVDITYAEYFEMSVRLAEAMKRYGLNTNHRIVVCS

        83                                                                              164
PpyLuc  ENSLQFFMPVLGALFIGVAVAPANDIYNERELLNSMNISQPTVVFVSKKGLQKILNVQKKLPIIQKIIIMDSKTDYQGFQSM
BgLuc   ENSLQFFMPVLGALFIGVAVAPANDIYNERELLNSMGISQPTVVFVSKKGLQKILNVQKKLPIIQKIIIMDSKTDYQGFQSM

        165                                                                             246
PpyLuc  YTFVTSHLPPGFNEYDFVPESFDRDKTIALIMNSSGSTGLPKGVALPHRTACVRFSHARDPIFGNQIIPDTAILSVVPFHHG
BgLuc   YTFVTSHLPPGFNEYDFKPESFDRDKTIALIMNSSGSTGLPKGVALPHRALCVRFSHARDPIFGNQIKPDTAILSVIPFHHA

        247                                                                             328
PpyLuc  FGMFTTLGYLICGFRVVLMYRFEEELFLRSLQDYKIQSALLVPTLFSFFAKSTLIDKYDLSNLHEIASGGAPLSKEVGEAVA
BgLuc   FGMSTTLGYLICGFRVVLMYRFEEELFLRSLQDYKIQSALLVPTLFSFLAKSTLIDKYDLSNLHEIASGGAPLSKEVGEAVA

        329                                                                             410
PpyLuc  KRFHLPGIRQGYGLTETTSAILITPEGDDKPGAVGKVVPFFEAKVVDLDTGKTLGVNQRGELCVRGPMIMSGYVNNPEATNA
BgLuc   KRFHLPGIRQGYGLTETTSAILITPKGDDKPGAVGKVVPFFEAKVVDLDTGKTLGVNQRGELCVRGPMIMSGYVNNPEATNA

        411                                                                             492
PpyLuc  LIDKDGWLHSGDIAYWDEDEHFFIVDRLKSLIKYKGYQVAPAELESILLQHPNIFDAGVAGLPDDDAGELPAAVVVLEHGKT
BgLuc   LIDKDGWLHSGDIAYWDEDEHFFIVDRLKSLIKYKGYQVAPAELESILLQHPNIRDAGVAGLPDDDAGELPAAVVVLEHGKT

        493                                                     550
PpyLuc  MTEKEIVDYVASQVTTAKKLRGGVVFVDEVPKGLTGKLDARKIREILIKAKKGGKSKL
BgLuc   MTEKEIVDYVASQVTTAKKLRGGVVFVDEVPKGLTGKLDARKIREILIKAKKGGKI--
```

Figure S1. Amino acid alignment of the BgLuc and PpyLuc luciferases. The red highlighted residues indicate the mutated amino acids.

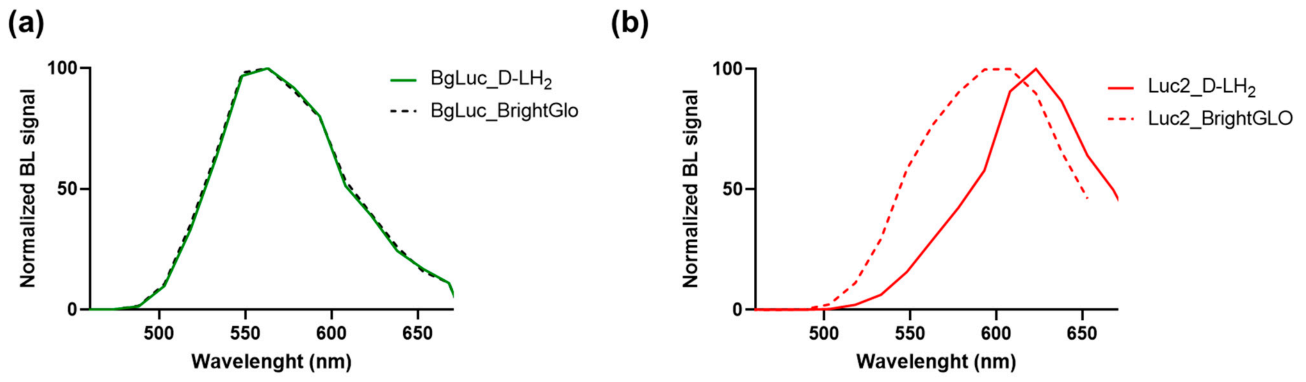

Figure S2: Emission spectra of (a) BgLuc and (b) Luc2 luciferase in HEK293T cells obtained with D-LH$_2$ substrate in citrate buffer (1.0 mM, pH 5.0) (solid line) and BrightGlo substrate (dotted line).

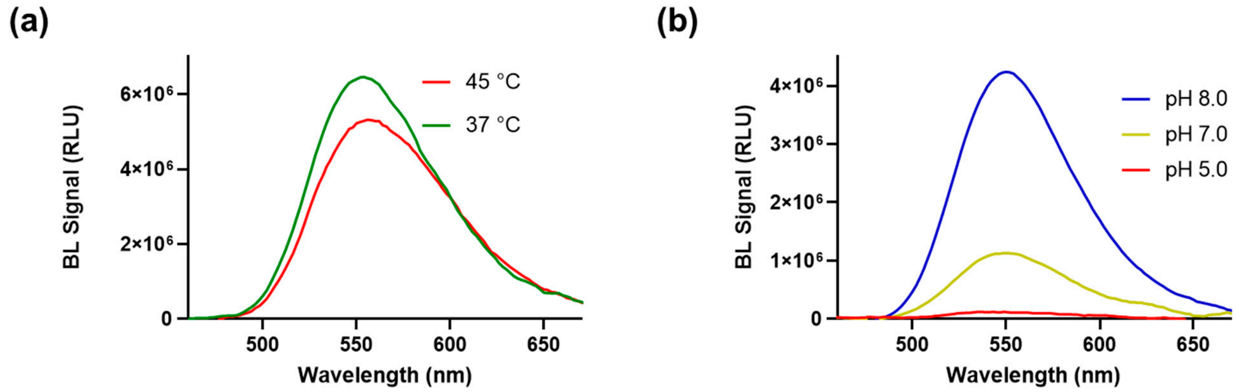

Figure S3: BgLuc mutant emission spectra obtained (a) at different temperatures and (b) at different pH with the commercial BrightGlo substrate.
